# First genome assembly and annotation of *Sanghuangporus weigelae* uncovers its medicinal functions, metabolic pathways, and evolution

**DOI:** 10.3389/fcimb.2023.1325418

**Published:** 2024-01-09

**Authors:** Can Jin, Jin-Xin Ma, Hao Wang, Lu-Xin Tang, Yi-Fan Ye, Xin Li, Jing Si

**Affiliations:** Institute of Microbiology, School of Ecology and Nature Conservation, Beijing Forestry University, Beijing, China

**Keywords:** Sanghuang, medicinal properties, secondary metabolites, cytochromes P450, carbohydrate-active enzymes, evolution

## Abstract

*Sanghuangporus*, also known as “Sanghuang” in China, is a well-known genus of traditional Chinese medicinal macrofungi. To make more effective use of *Sanghuangporus* resources, we completed the first genome assembly and annotation of a monokaryon strain of *S. weigelae* in the present study. A 33.96-Mb genome sequence was assembled as 13 contigs, leading to prediction of 9377 protein-coding genes. Phylogenetic and average nucleotide identity analyses indicated that the *S. weigelae* genome is closely related to those of other *Sanghuangporus* species in evolutionary tree, which clustered in one clade. Collinearity analysis revealed a high level of collinearity of *S. weigelae* with *S. baumii*, *S. vaninii*, and *S. sanghuang*. Biosynthesis pathways potentially involved in medicinal properties, including terpenoid and polysaccharide synthesis, were identified in *S. weigelae*, while polysaccharides were identified as the main medicinal metabolites in *S. weigelae*, with flavonoids more important in *Sanghuangporus* than other medicinal mushroom groups. Genes encoding 332 carbohydrate-active enzymes were identified in the *S. weigelae* genome, including major glycoside hydrolases and glycosyltransferases predicted, revealing the robust lignocellulose degradation capacity of *S. weigelae*. Further, 130 genes, clustered in seven classes were annotated to encode cytochromes P450 in the *S. weigelae* genome. Overall, our results reveal the remarkably medicinal capacity of *S. weigelae* and provide new insights that will inform the study of evolution and medicinal application of *S. weigelae*. The data are a reference resource for the formulation of scientific and rational ecological protection policies for *Sanghuangporus* species.

## 1 Introduction

*Sanghuangporus* is a traditional Chinese medicine that has been used in China to prevent and treat diseases for thousands of years (Dai and Cui, 2014). The long history of consumption and medicinal use of *Sanghuangporus* has brought health benefits for Chinese people and led to development of a unique traditional Chinese medicine culture surroundings *Sanghuangporus*, in the ancient ways of the Yellow River (Zhou et al., 2016). As a valuable class of medicinal mushroom, *Sanghuangporus* species are comparable with the famous Chinese medicine “DongchongXiacao” (*Ophiocordyceps sinensis*) (Wu et al., 2016). In the Compendium of Materia Medica compiled by Li Shizhen, it is recorded in Chinese that “Sanghuang” can “Li wu zang, Xuan chang qi”, meaning that it can benefit the five internal organs of the body (heart, spleen, liver, lungs, and kidneys) and promote gastrointestinal function (Huang et al., 2023). Separate from Chinese traditional medicine, *Sanghuangporus* species have been shown to be effective for treating neurodegenerative diseases, such as Parkinson’s and Alzheimer’s (Fang et al., 2020). Further, there is evidence that *Sanghuangporus* mycelia secrete high levels of bioactive substances, including polysaccharides, flavonoids, polyphenols, pyrones, terpenes, proteins, lipids, alkaloids, minerals, vitamins, and other substances (Wang X. T., et al., 2023). These bioactive compounds have prophylactic and therapeutic benefits, including tumor suppression, antioxidant, bacteriostatic, anti-inflammatory, blood glucose, liver protection, and immune regulation properties (Cai et al., 2019; Wei et al., 2023).

In recent years, the utility of traditional Chinese medicine, and *Sanghuangporus* in particular, has attracted increasing attention in spheres from folklore to scientific and industrial fields (Zheng et al., 2023). There are 18 known species of *Sanghuangporus*, ten of which are widely distributed in China, including *S. weigelae*, which mainly grows in subtropical warm temperate regions, at the base of or inside wood; the fruiting body is usually flat and inverted, with a light brown surface, and small amounts are available for sale on the market (Wu et al., 2016). *S. weigelae* ranks the second among eight *Sanghuangporus* strains with the highest polysaccharide yields (Wang H. et al., 2023), along with species such as *S. sanghuang*, *S. vaninii*, and *S. baumii*. Further, *S. weigelae* has superior 1,1-diphenyl-2-picrylhydrazyl (DPPH) radical scavenging ability, triterpenoid content, and ascorbic acid content. These results are consistent with those of an important study (Meng et al., 2016), demonstrating that, in liquid culture, *S. weigelae* has significantly higher capacity to generate polyphenols, malonaldehyde content, superoxide dismutase activity, total antioxidant capacity, and DPPH radical scavenging ability than that of *Perenniporia robiniophila*, as well as slightly higher capacity than *S. sanghuang*. Hence, *S. weigelae* is undoubtedly an important traditional Chinese medicinal resource.

With advances in DNA sequencing technologies, medicinal studies of wood-inhabiting macrofungi have gradually generated genome sequences, in addition to data on the metabolites produced (Kiss et al., 2019). Comparison of fungal genomes can reveal important information on the production of various bioactive compounds and metabolic pathways (Min et al., 2018). Genome assemblies of several Sanghuangporus mushrooms have recently been reported, including *S. sanghuang* (Jiang et al., 2021), *S. vaninii* (Song et al., 2021), and *S. baumii*, which are closely related to *S. weigelae*, and have generated several scaffold-scale genome sequences. Other medicinal mushrooms, such as *Ganoderma sinense*, *Pleurotus giganteus*, and *Oudemansiella raphanipes*, have been also subjected to whole genome sequencing. This sequencing and analysis of various fungi genomes has improved understanding of fungal growth and development. Nevertheless, data on *Sanghuangporus* on genome and transcriptome sequences are largely lacking, and the lack of a complete *S. weigelae* genome sequence has impeded deeper understanding of the mechanisms related to biosynthesis of bioactive compounds, such as terpenoids and polysaccharides, hindering further studies based on genome editing. Genome editing techniques have the potential to overcome oxidation issues in mushrooms, creating opportunities for developing improved strains. Only three whole genome sequences of *Sanghuangporus* species are available, which could hinder application of the unique medicinal properties of each *Sanghuangporus* species, delaying further applications for permissions to develop commercial products (Brandenburger et al., 2018).

Here, to promote the medicinal use and industrial development of *S. weigelae* from a genomic perspective, we assembled and annotated the high-level genome of *S. weigelae* and compared it with other related genomes. Besides information for medicinal application, our genomic analyses, particularly comparisons with genomes of other related fungal species, also reveal evolutionary information regarding the *Sanghuangporus* genus. Our genomic analyses will help to elucidate the medicinal properties, and biosynthetic pathways of these medicinal mushrooms, which will further facilitate their medicinal use and commercial development.

## 2 Materials and methods

### 2.1 Strain culture and DNA isolation

**Figure 1:**
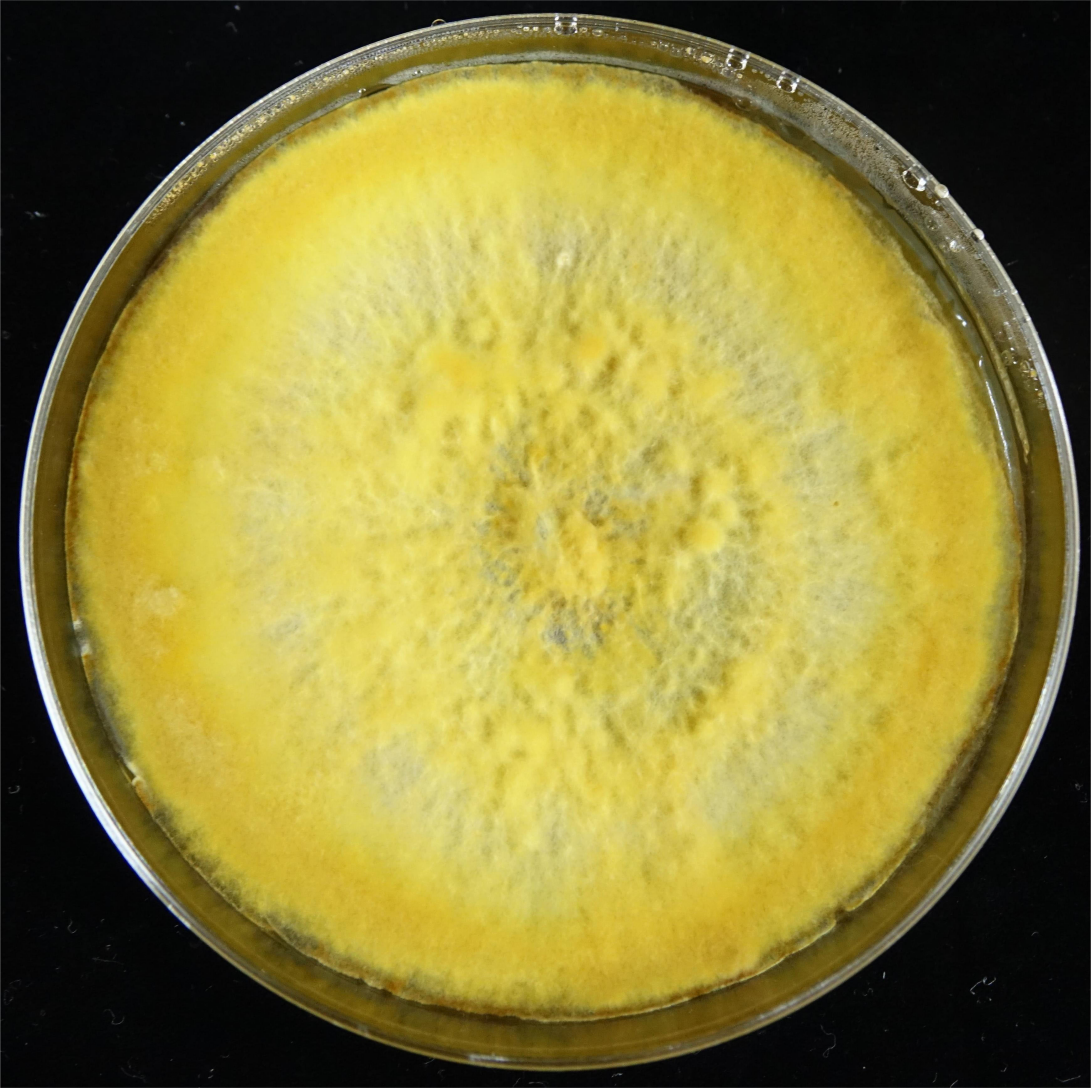
Monokaryotic mycelia of *Sanghuangporus weigelae*.

The monokaryotic strain of *S. weigelae* used in this study was isolated from a wild fruiting body collected from the fallen trunk of a *Weigela florida* specimen in Jinfoshan Forest Park, Chongqing, and has been deposited at Beijing Forestry University. Mycelia were harvested after growing on sterile cellophane covering potato dextrose agar (PDA) culture medium plates at 28°C for 5−7 d. Mycelia were collected from the liquid medium, packaged in aluminum paper, frozen in liquid nitrogen, and stored at -80°C for DNA extraction (**Figure 1**). *S. weigelae* was inoculated on PDA plates covered with cellophane. Mycelia were scraped from the cellophane after they covered the entire plate. High-quality DNA was extracted using the QIAGEN^®^ Genomic kit.

### 2.2 Genome sequencing and assembly

Libraries were constructed and raw sequencing data were obtained by single-molecule real-time fluorescence DNA sequencing using a Pacbio Sequel series sequencer (Wick et al., 2019). MGISEQ2000 platform genome sequencing data were processed using fastp v.0.20.0. Sequel and Sequel II downstream data were obtained using Pacbio proprietary Smrtlink software at the Nextomics Biosciences Co., Ltd (Wuhan, China), which uses high quality region finder to identify the longest region of a singly loaded enzyme that maintains its activity, and signal noise ratio to filter low quality regions and obtain subreads. Downlinked data had no single-base quality values and all base quality values were recorded, to maintain format integrity, but were not of practical significance. The read quality of reads that passed the filter was set at 0.8, and those that did not pass were labeled 0. For quality control, of HiFi reads, subreads were converted to HiFi reads using CCS software, and reads > 1000 bp filtered as pass reads, which were directly used for assembly. Since HiFi read sequences are highly accurate (≥ 99%), they are suitable for genome assembly using Hifiasm (parameter: -n 5). Subsequently, second-generation data were filtered using fastp (-n 0), and the genome polished using the filtered second-generation data. Four iterations of correction with Nextpolish were conducted to obtain the final genome sequence.

### 2.3 Gene prediction and annotation

Gene structure prediction was performed using a combination of three methods: *ab initio* prediction using AUGUSTUS (Stanke et al., 2008), homology-based prediction with GeMoMa (Keilwagen et al., 2016), and transcriptome prediction using PASA (Haas et al., 2003). The results were integrated with EvidenceModeler software (Haas et al., 2008), and final structural annotation were obtained by removing genes containing transposable elements using TransposonPSI (Urasaki et al., 2017). Interspersed repetitive sequences were predicted using RepeatMasker (Bedell et al., 2000). Tandem repeats were analyzed using GMATA (Wang and Wang, 2016) and Tandem Repeats Finder (Benson, 1999). tRNA genes were predicted using tRNAscan-SE (Lowe and Eddy, 1997). Gene functions were predicted with reference to these databases which were downloaded from the official web site and localized to compare and annotate the whole genome: Gene Ontology (GO) database (http://geneontology.org/), Kyoto Encyclopedia of Genes and Genomes (KEGG) database (https://www.kegg.jp/), Eukaryotic Orthologous Group (KOG) database (https://www.creative-proteomics.com/services/kog-annotation-analysis-service.htm), Non-Redundant Protein (NR) database (https://www.ncbi.nlm.nih.gov/prote-in), SwissProt database (https://www.uniprot.org/), Pfam database (http://pfam.xfam.org/), Fungal Cytochrome P450 (CYP) database (http://p450.riceblast.snu.ac.kr/cyp.php), and Carbohydrate-Active Enzyme (CAZyme) database (http://www.cazy.org). The whole genome predicted coding genes were aligned with these databases with cut-off values of E-value ≤ 1 × 10^-5^, identity ≥ 40%, and coverage ≥ 40% by DIAMOND (Buchfink et al., 2015). When a single gene retrieved from the database with more than one result meeting the cut-off values, the gene was annotated by the best score. The antiSMASH program (Medema et al., 2011) with default parameters was employed to predict gene clusters encoding secondary metabolites.

### 2.4 Comparative genomics analysis

Pairwise average nucleotide identity (ANI) values between genomes were determined using FastANI software (Jain et al., 2018). To explore the dynamics of speciation in *Sanghuangporus*, genome sequences of *S. weigelae*, *S. sanghuang*, *S. vaninii*, and *S. baumii* were aligned pairwise using MCScanX software, based on location information from *Sanghuangporus* GFF3 files (Wang et al., 2012). Based on the resulting blocks, a genomic synteny map among the four species was drawn using TBtools (Chen et al., 2020) supported in the jcvi package in Python 3. To identify the differences and similarities in medicinal applications of *Sanghuangporus* with a uniform standard, the genome sequences of *S. sanghuang*, *S. baumii*, and *S. vaninii* were reannotated using the same pipeline. Then, genome structure and protein-coding genes related to medicinal use were compared among these four species. In addition, the numbers of genes encoding various families of CAZymes in the four species were clustered in heatmaps using TBtools v.2.0, using the log scale option. To explore the evolutionary dynamics of *S. weigelae*, the genome sequences of 22 additional fungal species were downloaded from the National Center for Biotechnology Information (NCBI) (https://www.ncbi.nlm.nih.gov/genbank/) for phylogenomics (**Supplementary Table S1**). Single-copy orthologous genes from the 23 fungal species were inferred using OrthoFinder software (Emms and Kelly, 2019) with the mafft option for subsequent multiple sequence alignment. The species tree was visualized using iTOL software (Zhou et al., 2023). Two Ascomycetous species (*Neurospora crassa* and *Tuber melanosporum*) were selected as outgroup taxa from a further 21 Basidiomycetous species (**Supplementary Table S1**).

## 3 Results

### 3.1 Genome sequence assembly and annotation

**Figure 2:**
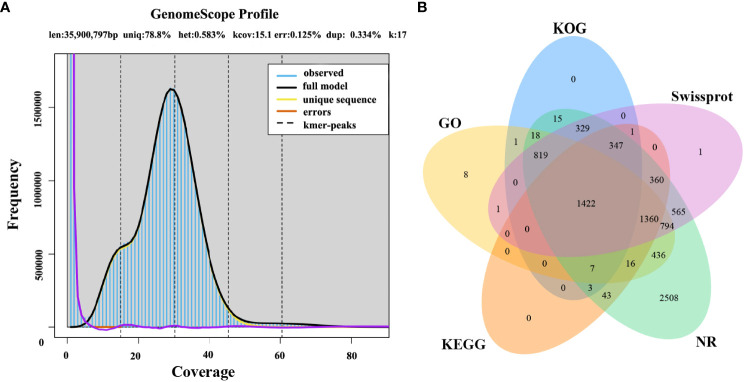
**(A)** Histogram of the depth distribution of *Sanghuangporus weigelae* sequencing. **(B)** Genes were annotated using information from five databases.

The first whole genome sequence of the medicinal fungus *S. weigelae* was generated using the PacBio Sequel II platform. Clean reads were obtained and used for K-mer analysis and genome polishing. GenomeScope v.1.0 was used to generate a histogram of sequencing depth distribution (k = 17) (**Figure 2A**). A single K-mer coverage peak was observed and the heterozygosity rate was 0.58%. The integrity of the genome was evaluated using BUSCO software (Simão et al., 2015) and determined to be 94.99%. A 33.96-Mb genome sequence was assembled from 10267 Mb raw data and mapped to 13 contigs, with a GC content of 47.93%. Of the 13 contigs, the longest was 4.49 Mb, while N50 length was 2.77 Mb; specific sequence length distribution data are presented in **Table 1**; **Supplementary Figure S1**.

**Table 1:** Genome assembly and features of *Sanghuangporus weigelae*.

Contig	Characteristic	Genome	Characteristic
Total number	13	Genome assembly (Mb)	33.96
N50 length (Mb)	2.77	Number of protein-coding genes	9377
Max length (Mb)	4.49	Average length of protein-coding genes (bp)	2061
Coverage (%)	99.99	Average CDS length (bp)	1521
GC content (%)	47.93	ncRNAs	142

The size and GC content of the *S. weigelae* genome were similar to those of other *Sanghuangporus* species; it was slightly larger than the *S. baumii* and *S. sanghuang* genomes, but smaller than that of *S. vaninii*. Analysis of GC skew did not reveal an obvious distribution pattern across the whole genome. Repeat sequences were identified using RepeatMasker, based on homology alignment and *ab initio* prediction, and accounted for 16.91% of the *S. weigelae* genome (**Table 2**; **Supplementary Figure S2**). The majority of repetitive sequences were long terminal repeats (LTR) (12.22%), where 1.62% and 1.16% of repeat elements were DNA transposons and miniature inverted-repeat transposable elements (MITEs). Long interspersed nuclear elements (LINEs) and short interspersed nuclear elements (SINEs) made up 0.06% and 0.01% of the *S. weigelae* genome, respectively.

**Table 2:** Repeat element analysis in the *Sanghuangporus weigelae* genome.

Repeat elements	Number of elements	Repeat size (bp)	Percentage of sequence (%)
LTR/Copia	699	636541	1.87
LTR/Gypsy	1914	1894577	5.58
LTR/others	1360	1619757	4.77
LINE	357	202643	0.60
SINE	29	5042	0.01
DNA/TcMar	45	76751	0.23
DNA/TcMar-Ant1	74	98959	0.29
DNA/others	114	28172	0.08
MITE	1215	392688	1.16
RC	1	74	0.00
Tandem repeats	2429	91071	0.27
Simple repeats	153	22230	0.07
Low complexity	2	418	0.00
Unclassified	2012	672925	1.98
Total	10404	5741848	16.91

A total of 9377 protein-coding genes were predicted (mean length, 2061 bp; mean coding sequences (CDS) length, 1521 bp) (**Table 1**), while 142 non-coding RNAs (ncRNAs) were predicted, accounting for 0.02% of the whole genome sequence. Of the identified genes, 9042 (96.43%) and 5999 (63.98%) (**Figure 2B**) were annotated in the NR and SwissProt databases, respectively. The completeness of *S. weigelae* gene prediction was also evaluated using BUSCO software with fungi_odb10. Together, these data indicate that the *S. weigelae* genome sequence assembly is high quality.

### 3.2 Terpenoid biosynthesis

In total, 16 genes encoding key enzymes involved in terpenoid backbone biosynthesis via the mevalonate (MVA) pathway were identified in *S. weigelae* genome sequence (**Supplementary Figure S3**). Among them, protein-*S*-isoprenylcysteine *O*-methyltransferase was encoded by a triple-copy gene, and both farnesyl diphosphate synthase and protein farnesyltransferase subunit *β* were encoded by double-copy genes, while the other 12 enzymes were encoded by single-copy genes (**Supplementary Table S2**). In addition to the 16 key enzymes in the MVA pathway, genes indirectly related to terpenoid biosynthesis were also identified: one gene (ID: C02.848) encoding farnesyl-diphosphate farnesyltransferase, one gene (ID: C02.604) involved in the biosynthesis of sesquiterpenoids and triterpenoids (**Supplementary Figure S4**), and the *β*-apo-4′-carotenal oxygenase, encoded by a single copy gene (ID: C04.738) involved in carotenoid biosynthesis. For example, the mevalonate kinase existed in terpenoid backbone biosynthesis in *S. sanghuang* but not in *S. weigelae.* The diphosphomevalonate decarboxylase, hydroxymethylglutaryl-CoA reductase, and protein-*S*-isoprenylcysteine *O*-methyltransferase were only found in *S. weigelae*. The four species of *Sanghuangporus* had a comparable number of genes involved in terpenoid backbone synthesis pathways (15 genes in *S. baumii*; 14 genes in *S. sanghuang*; 15 genes in *S. vaninii*; 18 genes in *S. weigelae*) (**Table 3**).

**Table 3:** Comparative genomics analyses of Sanghuangporus baumii, S. sanghuang, S. vaninii, and S. weigelae.

Characteristic		*S. baumii*	*S. sanghuang*	*S. vaninii*	*S. weigelae*
Reference		Jiang et al., 2021; Shen et al., 2023			This study
Genome structure	Genome size (Mb)	31.64	33.34	34.52	33.96
	Number of contigs	339	26	37	13
	N50 length of contig (Mb)	0.18	2.06	2.02	2.77
	Protein-coding genes	8455	8278	11310	9377
	GC content (%)	47.25	48.04	47.95	47.93
Numbers of genes involved in medicinal metabolite pathways	Terpenoid backbone biosynthesis	15	14	15	18
	Polysaccharide biosynthesis	40	41	46	46
	Flavonoid biosynthesis	6	8	7	0
Numbers of genes encoding CAZymes	CBM	39	38	38	38
	CE	15	23	18	23
	GH	155	162	160	169
	GT	54	62	61	50
	PL	5	9	8	6
	AA	45	52	53	46
	Total	313	346	338	332
Numbers of genes encoding CYPs	B-class P450	1	0	0	0
	Cytochrome P450	8	12	9	8
	E-class P450, CYP2D	1	1	1	1
	E-class P450, group I	68	66	80	65
	E-class P450, group IV	7	8	14	20
	P450, CYP52	5	5	6	5
	Pisatin demethylase-like	4	6	2	8
	Undetermined	18	25	24	23
	Total	112	121	136	130
Numbers of secondary metabolite gene clusters	TS	12	10	11	9
	T1PKS	1	4	1	3
	NRPS	3	1	3	4
	Other molecules	2	1	1	3
	Total	18	16	16	19

### 3.3 Polysaccharide biosynthesis

A total of 19 enzymes, encoded by 48 genes, involved in the biosynthesis of polysaccharides (starch and sucrose metabolism) were identified in *S. weigelae* genome (**Supplementary Figure S5**; **Supplementary Table S3**). Most of these enzymes were encoded by single- or double-copy genes, while the endoglucanase, glucan 1,3-*β*-glucosidase and cellulose 1,4-*β*-cellobiosidase, and *β*-glucosidase were encoded by four, five, six, and nine-genes, respectively. The four species of *Sanghuangporus* had similar numbers of genes involved in polysaccharide biosynthesis pathways, namely 40 genes in *S. baumii*, 41 genes in *S. sanghuang*, 46 genes in *S. vaninii*, and 46 genes in *S. weigelae* (**Table 3**). The differences were focused on two genes encoding 1,3-*β*-glucosidase and seven genes encoding *β*-glucosidase. Among them, genes encoding polysaccharide biosynthesis were identified and remarkably higher in the genome of *S. weigelae* than those of *S. vaninii* and *S. sanghuang* (**Supplementary Table S3**).

### 3.4 Flavonoid biosynthesis

No key enzymes directly related to flavone and flavonoid biosynthesis pathways were identified in the *S. weigelae* genome; however, six putative genes involved in flavonoid compound biosynthesis were annotated, including P-type H^+^-ATPase, anthocyanin 3-*O*-6″-*O*-coumaroylglucoside: glucosyltransferase, anthocyanin 3-*O*-6″-*O*-coumaroylglucoside: glucosyltransferase, acetyl-CoA carboxylase, glutathione *S*-transferase, and flavonoid 3-*O*-glucosyltransferase (**Supplementary Table S4**).

### 3.5 Carbohydrate-active enzymes

**Figure 3:**
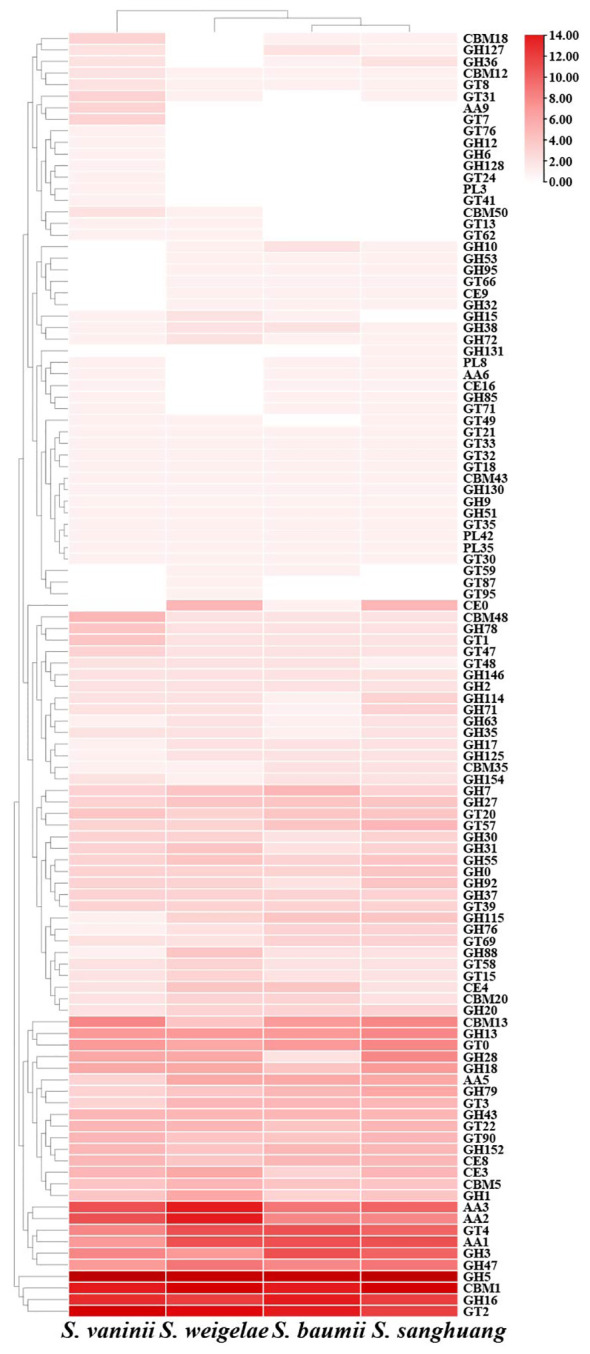
Heatmap of CAZyme families found in *Sanghuangporus vaninii, S. weigelae, S. baumii*, and *S. sanghuang*. The x and y axes represent species and CAZyme families, respectively. Boxes are colored according to log values of gene numbers encoding CAZyme families, where box color from white to red indicates an increase in gene numbers encoding CAZyme families.

CAZymes play important roles in drug metabolism, xenobiotic detoxification, and steroid biosynthesis. In *S. weigelae*, a total of 332 genes were predicated to encode six classes of CAZymes, including 38 genes encoding carbohydrate-binding modules (CBMs), 23 carbohydrate esterases (CEs), 169 glycoside hydrolases (GHs), 50 glycosyltransferases (GTs), 6 polysaccharide lyases (PLs), and 46 auxiliary activities (AAs) (**Table 3**). Among these six classes, GHs were encoded by the most genes and are mainly involved in degradation of celluloses (GH5 and GH7), hemicelluloses (GH43), pectins (GH28), chitins (GH18), and starches (GH31). Families encoded by > 10 genes included GH5 (23), CBM1 (18), GT2 (15), AA3 (14), AA2 (14), GH16 (12), GT4 (11), and AA1 (11). The total numbers of genes encoding CAZymes were similar among the four species of *Sanghuangporus* (*S. baumii* 313; *S. sanghuang* 346; *S. vaninii* 338), as was the number of genes encoding each of the six classes of CAZymes (**Table 3**). Only a few differences were detected at the level of gene families encoding CAZymes (**Figure 3**). Based on the numbers of genes belonging to different CAZyme families, *S. weigelae* appears to have a more similar strategy for use of woody substrates to that of *S. sanghuang* than that of *S. baumii*.

### 3.6 Cytochromes P450

**Figure 4:**
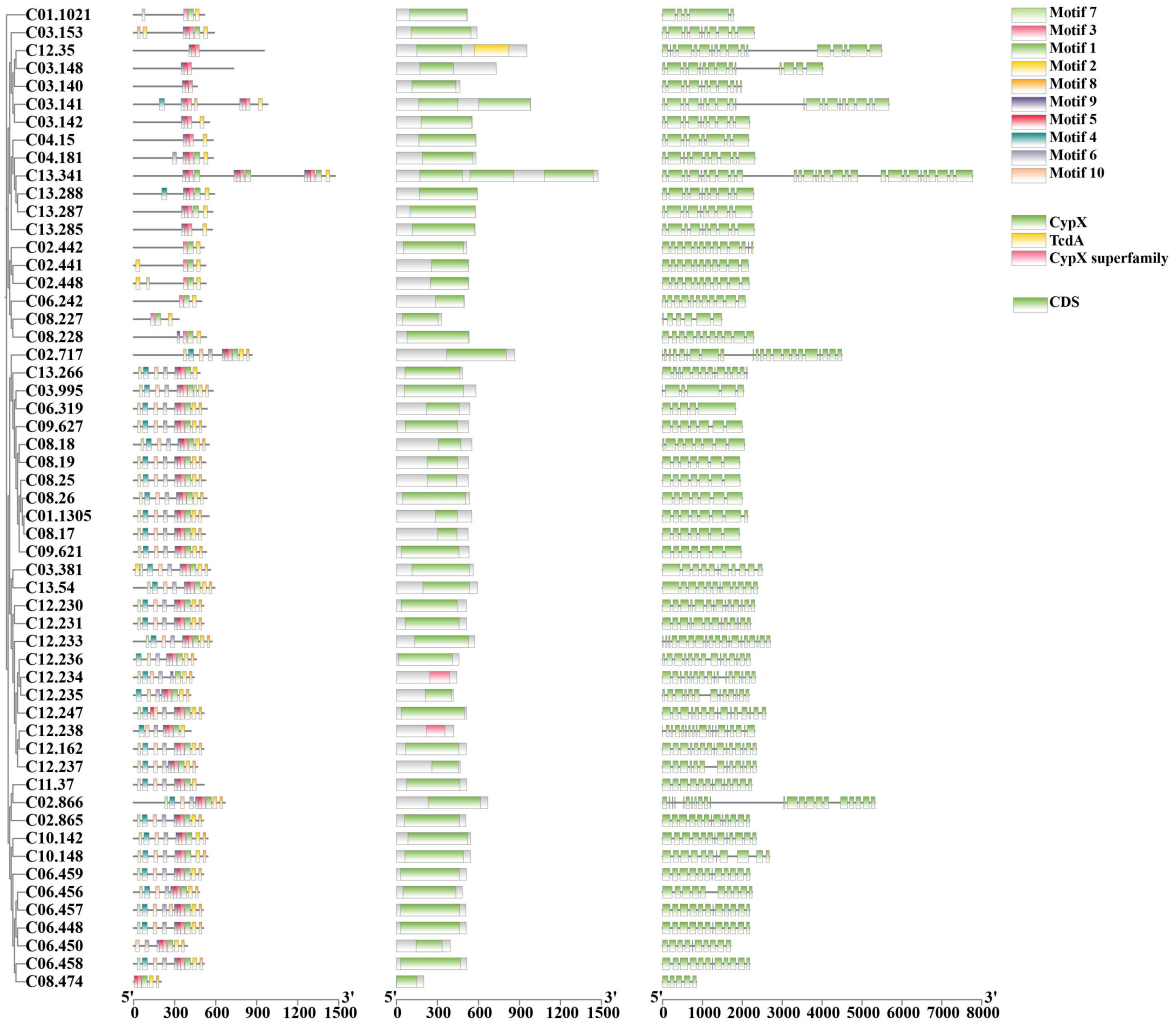
A combined plot of evolutionary tree, motif, structural domain, and gene structure of 55 genes with the conserved motifs of E-class P450, group I class of CYP family proteins.

CYPs are among the most important gene families in the fungal genome. A total of 130 genes were annotated to encode CYPs in *S. weigelae* (**Table 3**). Of these genes, eight were classified to encode CYPs belonging to the ‘Cytochrome P450’ class, one to the ‘E-class P450, CYP2D’ class, 65 to the ‘E-class P450, group I’ class, 20 to the ‘E-class P450, group IV’ class, five to the ‘P450, the CYP52’ class, and eight to the ‘pisatin demethylase-like’ class, while 23 genes were determined to encode CYPs not belonging to any known class. The ‘E-class P450, group I’ (**Figure 4**) and ‘E-class P450, group IV’ classes encoded by the most genes in *S. weigelae*, are involved in oxidation-reduction reactions, while the CYP classes encoded by the next largest number of genes are generally responsible for signal transduction of metabolic processes. The numbers of genes encoding CYPs in *S. weigelae* (130) did not differ significantly at the class level from those in the other three species *S. baumii* (112), *S. sanghuang* (121), and *S. vaninii* (136). The differences are focused on the numbers of genes encoding ‘E-class P450, group I’ class, with the highest in *S. sanghuang* (80), followed by other three species fluctuating around 65. The numbers of genes encoding other classes including ‘B-class P450’, ‘Cytochrome P450’, ‘E-class P450, CYP2D’, ‘E-class P450, group IV’, ‘P450, CYP52’, ‘Pisatin demethylase-like’, and ‘Undetermined’ were almost consistent (**Table 3**).

### 3.7 Gene clusters

Nineteen gene clusters were predicted in the *S. weigelae* genome, of which nine were identified to encode terpene synthases (TSs), three to encode iterative type I polyketide synthases (T1PKSs), four to encode nonribosomal peptide synthetases (NRPSs), and three to encode other molecules (**Table 3**). Among the four species of *Sanghuangporus*, the numbers of gene clusters involved in TS synthesis were similar, while *S. weigelae* had more gene clusters involved in T1PKS and NRPS synthesis (**Table 3**).

### 3.8 Comparative genomics

**Figure 5:**
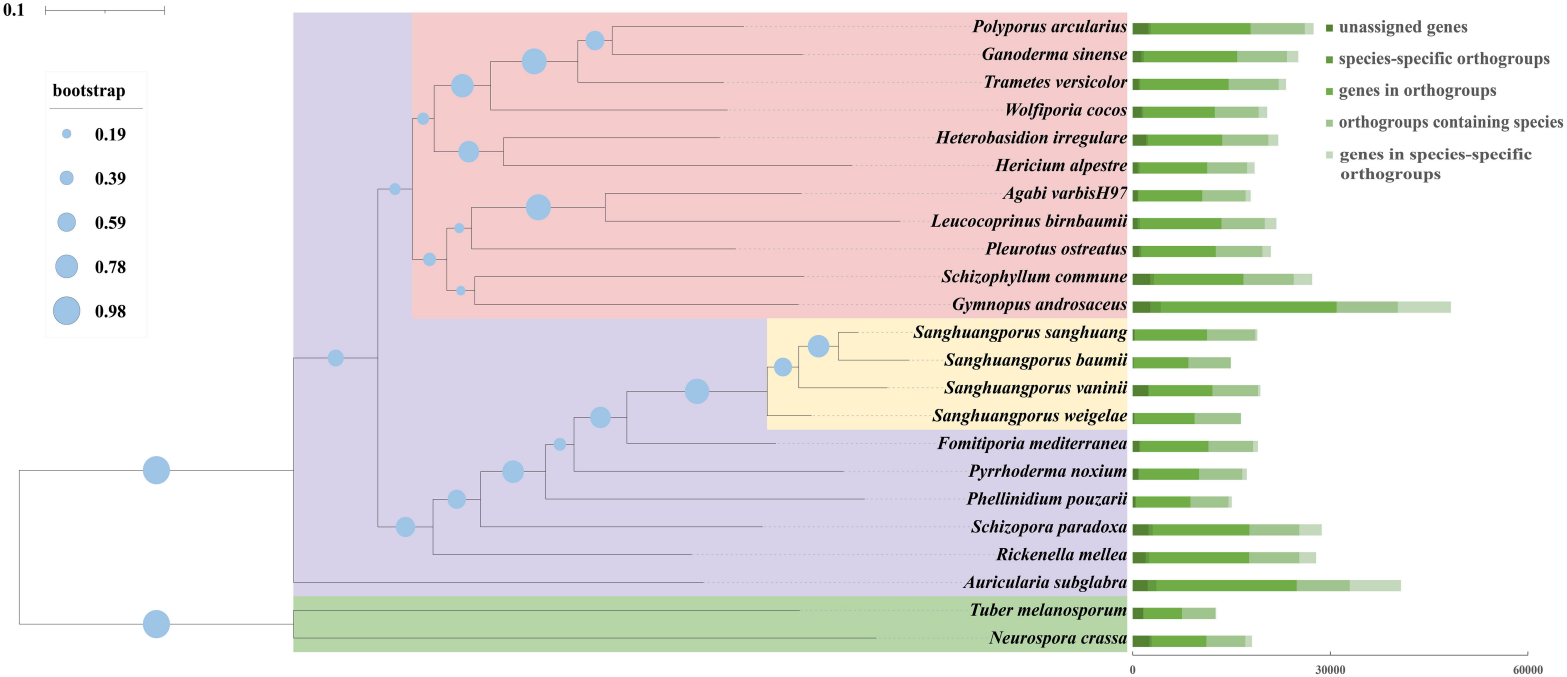
Comparison of genomes among *Sanghuangporus weigelae* and 22 other fungal species (20 Basidiomycetes and 2 Ascomycetes). Evolutionary relationship analysis and phylogenetic tree construction were conducted based on 451 single-copy orthologous genes using OrthoFinder. Single-copy orthologs were defined as orthologs present as a single-copy gene in all 23 species. Numbers of different orthologous gene types were calculated in each fungal species and are indicated by different colors. Tree scale = 0.1.

*Sanghuangporus* are rare medicinal mushrooms with long history of controversial species classification and are not easily distinguished from several species of similar brown rot fungi, which has seriously affected the use of *Sanghuangporus* as a medicinal resource. To assess the evolutionary relationships of *S. weigelae*, we conducted comparative analysis of the *S. weigelae* genome and those of 22 fully sequenced fungi (20 Basidiomycetes and 2 Ascomycetes). A total of 1647 orthologous groups, including 451 single-copy genes, were identified in all 22 studied fungal species. A phylogenetic tree constructed based on conserved single copy orthologous gene alignment showed that *S. weigelae* had a close evolutionary relationship with other *Sanghuangporus* species (**Figure 5**), particularly *S. vaninii*. Indeed, the fruiting bodies of the four species share similar characteristics, including sessile ascospores; field-grown ascospores, mostly superposed; fresh ascospores, corky; and sclerotized after drying.

Next, we analyzed orthogroups among the four *Sanghuangporus* species. The *S. weigelae* genome sequence had a higher similarity to that of *S. sanghuang* (90.32%), *S. baumii* (90.28%), and *S. vaninii* (89.73%); however, while 5412 orthologous groups were identified in the four *Sanghuangporus* species, *S. weigelae* shared more orthologous groups with *S. sanghuang* (125) and *S. vaninii* (102) than with *S. baumii* (32) (**Supplementary Figure S6**). In addition, *S. vaninii* had the largest number of unique orthologous groups (219), followed by *S. weigelae* (164) (**Supplementary Figure S6**). As shown in **Supplementary Figure S6**, *S. weigelae* and *S. sanghuang* shared 125 orthogroups, which were higher than those shared with *S. baumii* (32 orthogroups). *S. weigelae* and *S. sanghuang* have similar fruiting bodies. Therefore, we predict that these 125 orthogroups may be related to fruiting body shape and other properties. The underlying detailed molecular mechanisms require investigation by differential analysis of gene or protein expression in these species.

**Figure 6:**
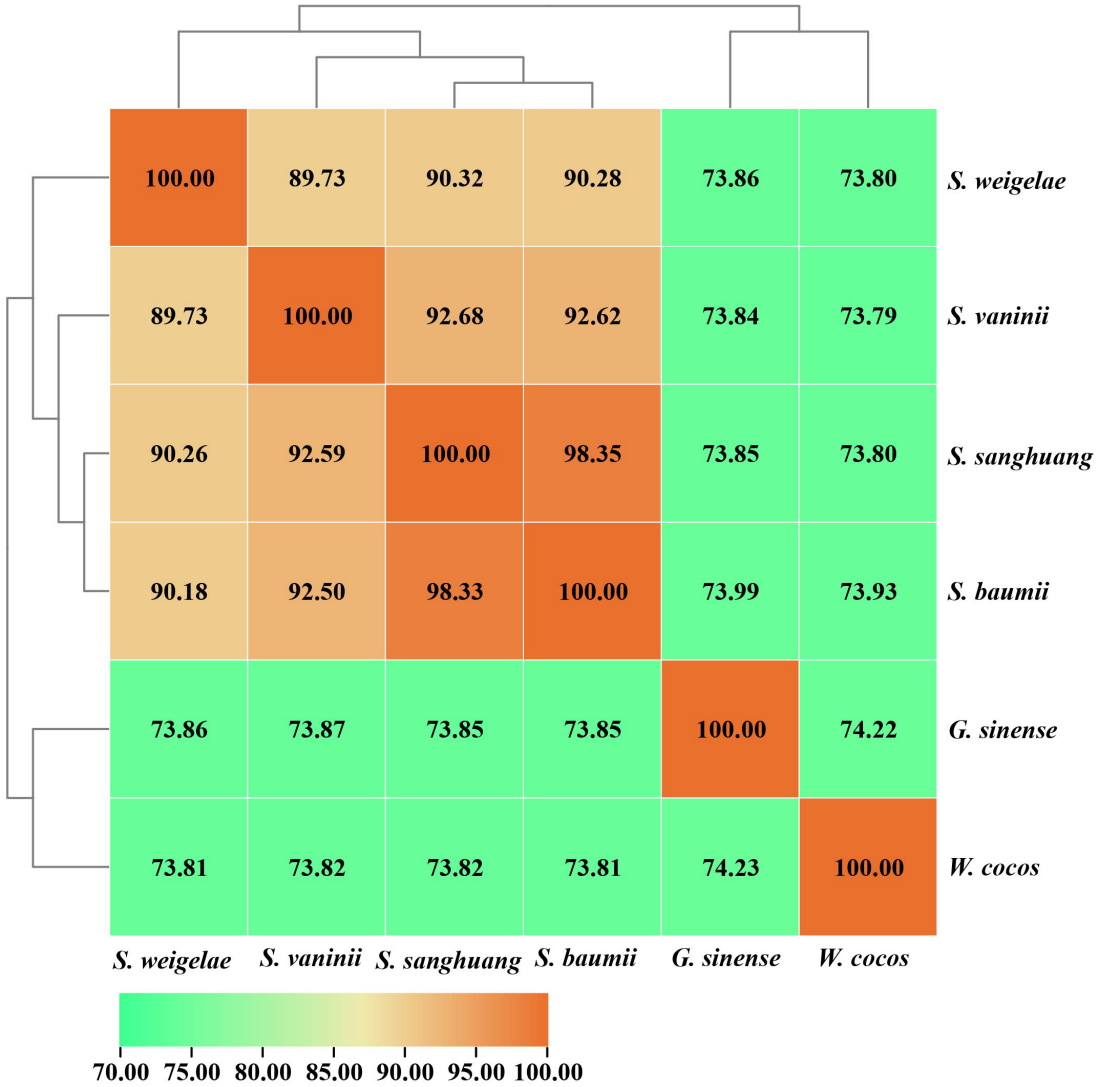
ANI values based on the fastANI algorithm generated-matrix for *Sanghuangporus*, *Ganoderma sinense*, and *Wolfifiporia cocos* genomes. Clustering was conducted using Euclidean distance matrix.

**Figure 7:**
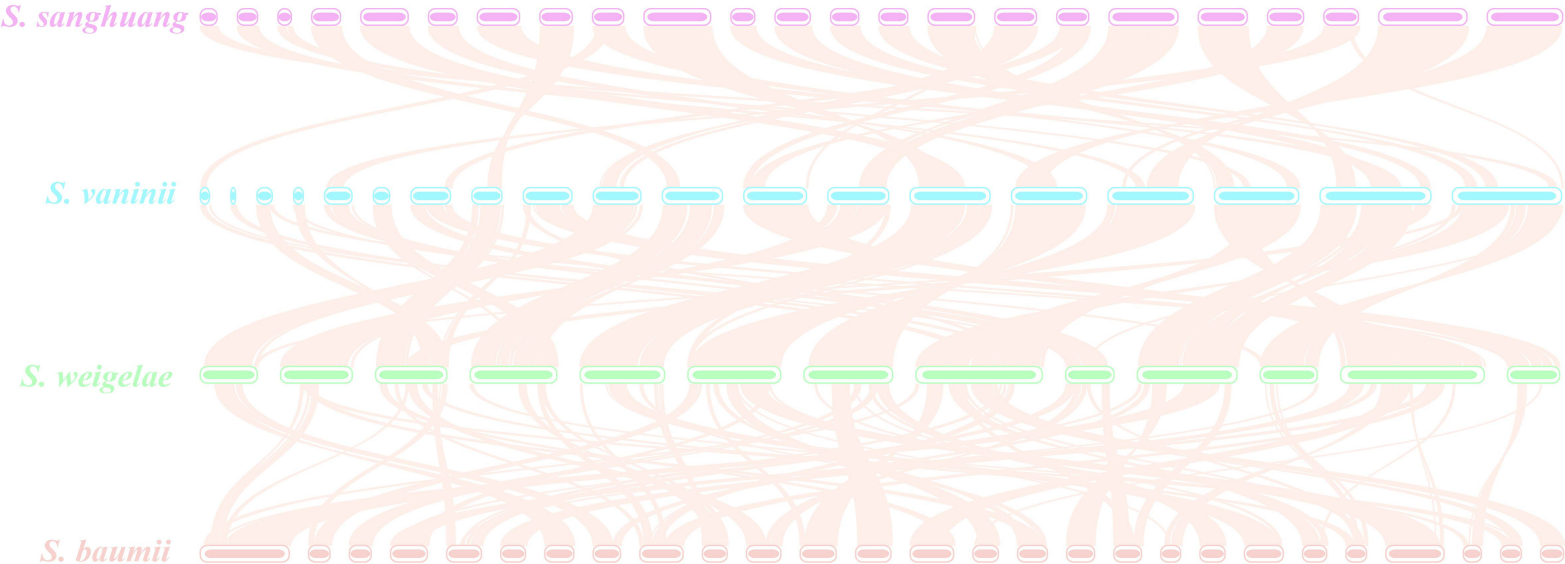
Genome collinearity among *Sanghuangporus sanghuang*, *S. vaninii*, *S. weigelae*, and *S. baumii*. Each line connects a pair of collinearity blocks between two genomes (*S. sanghuang* and *S. vaninii*, 16141 of collinear genes, 73.50%; *S. vaninii* and *S. weigelae*, 15797 of collinear genes, 78.28%; *S. weigelae* and *S. baumii*, 12794 of collinear genes, 71.53%).

The ANI analysis method facilitates high-resolution classification and is widely used for research. To further understand the evolutionary relationships among these mushrooms, ANI analysis was performed to estimate genomic differences and relatedness between *Sanghuangporus* species, *G. sinense*, and *Wolfifiporia cocos*. *Sanghuangporus* species in the species tree showed lower genomic similarities (73% to 75%) with *G. sinense* and *W. cocos*, but higher genomic similarities with one another (88% to 100%) (**Figure 6**). In summary, these results confirm that *S. weigelae* belong to the *Sanghuangporus* genus, consistent with its current classification. Collinearity analysis of *S. weigelae* and other three medicinal mushrooms, which have high levels of genome similarity, was performed using MCScanX software (**Figure 7**). The results revealed high levels of collinearity between *S. weigelae* and *S. sanghuang*. The connection of these contigs could be further confirmed using PCR or HiC technology.

## 4 Discussion

### 4.1 Cultural origins of *Sanghuangporus weigelae* genome sequencing

**Figure 8:**
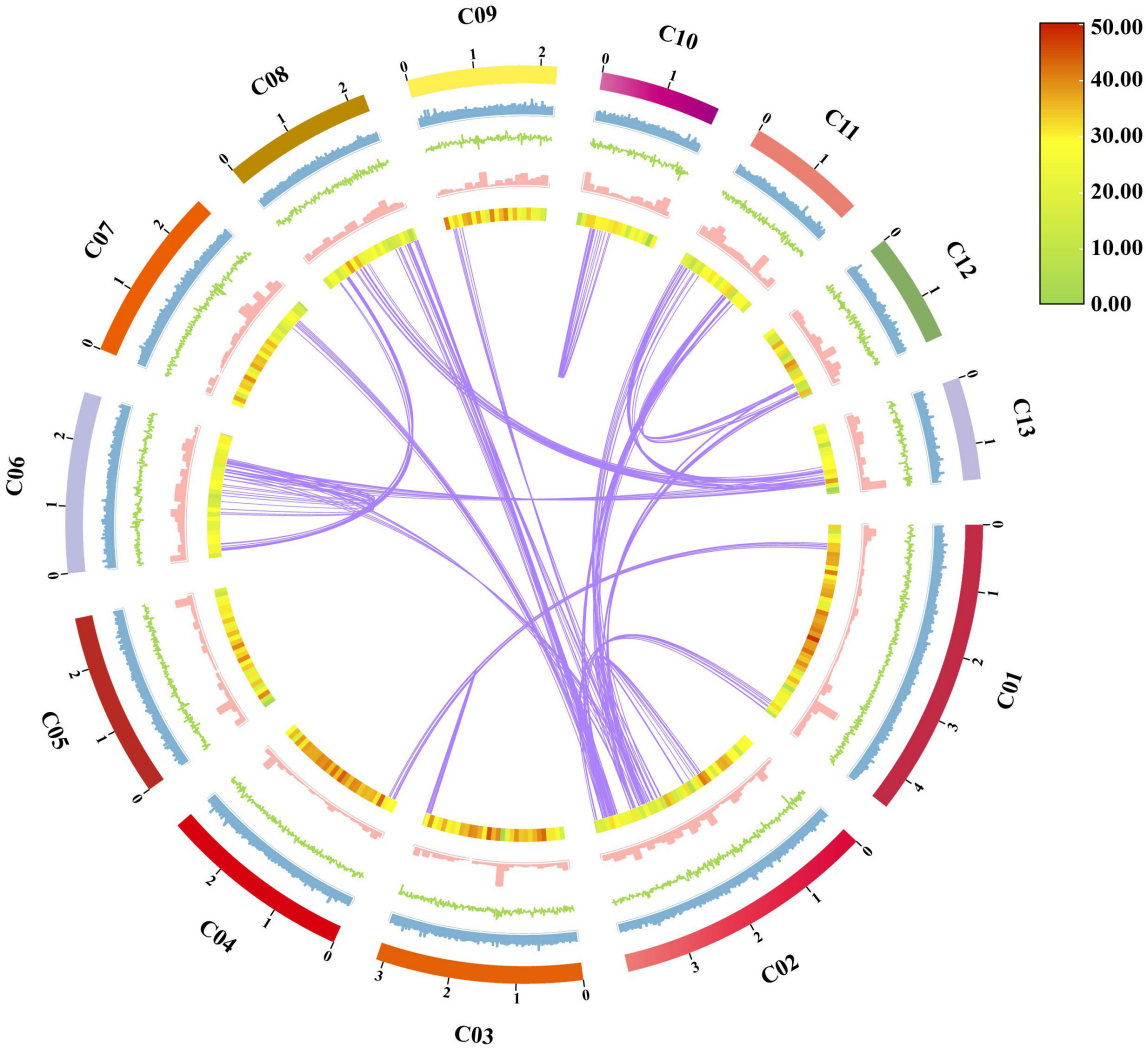
Characteristics of *Sanghuangporus weigelae* genomic assembly features. From outside to inside: (1) Contigs (> 1 Mb in length); (2) GC ratio, calculated as the percentage of G + C in 1 kb non-overlapping windows; (3) GC skew, calculated as the percentage of (G − C)/(G + C) in 1 kb non-overlapping windows; (4) Repeat sequence density per window; (5) Gene density per window; (6) Collinearity in the whole genome, calculated as 1e-5E-value, 5 number of hits, and 5 number of aligns connected by purple lines.

Chinese medicine, the treasure of ancient Chinese science (He et al., 2023), is also known as “the key to unlocking the treasury of Chinese civilization” (Bai et al., 2022), and has become an important part of cultural exchanges between China and other countries, spreading and gaining influence around the world, and as well as the promotion of exchanges and mutual understanding between the civilizations of the East and the West (Guo et al., 2023). “Sanghuang” (*Sanghuangporus*), a valuable traditional Chinese medicine, known as “forest gold” (Cao et al., 2020), can be compared with the Chinese medicines, “Lingzhi” (*Ganoderma lucidum*), “Zizhi” (*G. sinensis*), and “Fuling” (*W. cocos*), which have been the focus of intense research. Among *Sanghuangporus* species, *S. weigelae* is a medicinal mushroom used in traditional Chinese medicines (Wang et al., 2022; Zhang et al., 2022), and has been recognized as possessing important medicinal properties, such as polysaccharide yields, DPPH radical scavenging ability, and triterpenoid content. To date, the taxonomic status of 18 *Sanghuangporus* species have been described in the NCBI database, and 15 *Sanghuangporus* species are considered to be “Sanghuang”, with *S. weigelae* among those with superior pharmacological activity (Wang H. et al., 2023). As a potentially important medicinal fungal resource, the genome sequence of *S. weigelae* has not previously been published. In the current study, we generated the first whole genome sequence of *S. weigelae* (**Figure 8**) to provide theoretical support and basic research content to advance understanding of traditional Chinese medicine and the development of the macrofungal industry.

### 4.2 Characteristics of *Sanghuangporus weigelae* genome

Whole-genome similarity analysis is among the best ways to decipher strain taxonomy and generate genetic information on the molecular mechanisms underlying fungal growth and breeding (Yu et al., 2022). In this study, we conducted the whole genome sequencing to explore the evolutionary status and information about functional genes in *S. weigelae*. We assembled the *S. weigelae* genome into 13 contigs, with much higher quality sequencing data than those reported for the closely related species strains, *S. baumii*, *S. vaninii*, and *S. sanghuang*. Genome sizes were relatively similar among the four *Sanghuangporus* species, while the numbers of predicted protein-coding genes were much higher in *S. vaninii* and *S. baumii* (**Table 3**). ANI values indicated that *S. weigelae* and its closely related strains are interspecies (90 < ANI < 95) (Jain et al., 2018), demonstrating that high-quality genome sequencing of different species can help to clarify their phylogenetic and evolution status. To comprehensively analyze the relationships among *S. weigelae* and related species from other genera, 20 Basidiomycota and 2 Ascomycota fungal species were included in the phylogenetic analysis, which demonstrated that *S. weigelae* is closely related to *S. vaninii*, *S. sanghuang*, and *S. baumii*. All of these Agaricomycetes commonly have fruiting bodies, which are good resources for studying genomic changes underlying incomplexity levels in mushroom fruiting bodies (Coelho et al., 2017). Understanding the genetic basis of fruiting body evolution may directly contribute to the improvement of medicinal fungal culture and production. Furthermore, synteny analysis revealed high collinearity between the *S. weigelae* and *S. sanghuang* genomes, suggesting that *S. sanghuang* can likely serve as a reference mode for cultivation and breeding of *S. weigelae* during largescale industrialization.

### 4.3 Pharmacological properties of *Sanghuangporus weigelae* from a genomic perspective

Aqueous extracts of “Sanghuang” can induce apoptosis of cancer cells, as demonstrated by researchers from Japan and Korea, and its anticancer efficacy has been reported (Ayeka, 2018). The substances with anticancer effects are due to the polysaccharide components of the fruiting bodies, which enhanced by our new perspective on *Sanghuangporus* secondary metabolites (Seçme et al., 2018). Notably, terpenoids and polysaccharides are two major classes of chemical compounds among the medicinal metabolites in *Sanghuangporus* that exhibit multiple pharmacological properties (Cai et al., 2019). Among the secondary metabolite gene clusters, those encoding T1PKS and NRPS are frequently detected in filamentous fungi, whereas TS-encoding gene clusters are more common in Basidiomycetes (Shao et al., 2020). Consistently, the four *Sanghuangporus* species analyzed here contained more TS gene clusters that participate in terpenoid biosynthesis (**Table 3**). Triterpenoids extracted from *Sanghuangporus* species have high antioxidant activity in the presence of added fungal polysaccharide inducers or ultrasound stimulation (Cai et al., 2019). Genes of the conserved MVA pathway are involved in terpenoid skeleton biosynthesis. In the current study, we show that *S. baumii*, *S. sanghuang*, *S. vaninii*, and *S. weigelae* share similar genotypes and gene numbers annotated in the MVA pathway (**Table 3**), indicating that triterpenoids from these species have similar medicinal potential. More studies of the biological properties of triterpenoids in *S. baumii*, *S. sanghuang*, *S. vaninii*, and *S. weigelae* are warranted.

We also identified numerous essential genes related to secondary metabolites, which endow *S. weigelae* with biological activities that promote its survival in a specific environment and defense responses to pathogens. Genes involved in regulation of terpenes and NRPSs were found in the *S. weigelae* genome. Terpenes are among the largest groups of bioactive natural products identified and play crucial roles in the biological functions identified in wild edible mushrooms (Quin et al., 2014; Min et al., 2018; Liang et al., 2020). For example, triterpenes from *G. lucidum* have significant anti-tumor effects and their potential for anti-tumor application has been assessed in the context of cancer treatment (Li et al., 2020). *S. weigelae* has a large number of terpene synthesis genes relative to reports regarding those in other medicinal mushrooms (**Table 3**). NRPSs are considered key factors in host-pathogen interactions of medicinal mushrooms, yet their specific functions have not been identified (Oide and Turgeon, 2020; Ilyukhin et al., 2022). Further, no function of NRPSs in Basidiomycetes has been reported to date (Duan et al., 2022). The numbers of terpenes and NRPSs in *S. weigelae* may indicate potential for medicinal development of the pathways generating biologically active chemicals in *Sanghuangporus*, and further research is urgently needed to investigate this area.

Besides genes involved in triterpenoid biosynthesis, several genes involved in sesquiterpenoid synthesis were also annotated in the *S. weigelae* genome. The results were similar to those of previous studies, indicating that pathways potentially involved in biosynthesis of terpenoids other than triterpenoids occur in *Sanghuangporus* (Jiang et al., 2021). Notably, sesquiterpenoids were extracted from *Sanghuangporus* and showed antibacterial, antifungal, and cytotoxic activities (Rajachan et al., 2020). These results suggest that sesquiterpenoids may be among common bioactive secondary metabolites in *Sanghuangporus*.

Polysaccharides are the most widely studied class of compounds among fungal secondary metabolites (Meng et al., 2016; Zheng et al., 2017; Wang et al., 2021; Wang et al., 2022). Differences in molecular weight, branching configuration, conformation, and chemical modification of polysaccharides provide the basis for their diverse biological activities, such as antitumor, antioxidant, and anti-inflammatory effects (Maity et al., 2021). These medicinal functions have also been detected in *Sanghuangporus* (Jiang et al., 2021; Jiang et al., 2022). Here, for the first time, we identified a polysaccharide biosynthesis pathway in *S. weigelae*, namely the starch and sucrose metabolism pathway, which involved 48 genes (**Supplementary Figure S3**; **Supplementary Table S2**). Similar genes were revealed to be involved in polysaccharide biosynthesis pathways in *S. baumii*, *S. sanghuang*, and *S. vaninii* (**Table 3**). The higher numbers of genes involved in polysaccharide biosynthesis, relative to other medicinal pathways, in *Sanghuangporus* suggest that polysaccharides may be the major medicinal metabolites in *Sanghuangporus*. The dominant numbers of genes encoding the water-soluble 1,3-*β*-and 1,6-*β*-glucans, the most active immunomodulatory and antioxidant compounds (Shao et al., 2020), accounted for the pharmaceutical potential of *S. weigelae*. However, further experiments are expected and required to test this hypothesis.

Unlike terpenoids and polysaccharides, flavonoids have received little attention in mushrooms, partly because they comprise a low proportion of total metabolites. Only six related flavoprotein genes were identified in *S. weigelae*, consistent with previous findings that the absence of chalcone isomerase 1 in the flavonoid biosynthesis pathway suggests that this mushroom synthesizes flavonoids by a different mechanism from that in plants (Shao et al., 2020). Flavonoids in mushrooms also deserve to become the focus of increased attention in medicinal studies, as these gene clusters may confer favorable medicinal properties in *S. weigelae*. In addition, differential expression of genes associated with medicinal properties may be responsible for the variation in medicinal properties among *Sanghuangporus* species. Transcriptomic and metabolic data could help to address this question. Further explorative work including elucidation of the effects of number and diversity of genes encoding related enzymes on the medicinal values will assist in cultivation of medicinal mushrooms and development of health care products.

### 4.4 Specification and uniqueness in *Sanghuangporus*

A major reason for the lack of widespread and large-scale utilization of mushrooms is the shortage of mature fruiting bodies, and the corresponding practical solution to this restriction is artificial cultivation (Saini et al., 2022). Genomics data has potential to provide clues to facilitate the cultivation of *S. weigelae*. Recent study has shown that CAZymes are crucial for the growth and development of wood-inhabiting fungi and for them to thrive in environments rich in carbohydrates, particularly lignocellulose and cellulose (Sharma et al., 2022; He et al., 2023). In the *S. weigelae* genome, the main CAZymes were GHs, GTs, and AAs, while PLs, CEs, and CMBs were in the minority. The number of GH genes, the most abundant family in the *S. weigelae* genome, was four times than that of GT genes, possibly due to the fact that lignocellulose degradation capacity is necessary for *S. weigelae* survival, as most GH-related genes encode proteins involved in starch degradation. These data reveal that GH gene enrichment has contributed to the diversification of nutrient substrate utilization in *S. weigelae*. Moreover, compared with other mushrooms, *S. weigelae* has the most AA genes, which are important for lignin degradation. The CAZymes of *S. weigelae* were analyzed in comparison with those of *S. vaninii*, *S. baumii*, and *S. sanghuang*. In all four species, genes encoding GH5, GH16, AAs, and GH18 were relatively abundant (**Figure 3**). GH5, one of the largest GH families, historically known as “cellulase family A”, has a wide range of specificities, is extremely abundant in various ecological niches, and is often found encoded as part of microbial communities (Aspeborg et al., 2012). GH16 members are widely distributed in all areas of life, in which they play various biological roles, including in the degradation of xyloglucan (Viborg et al., 2019). AA9, a class of copper-dependent oxidases that act on crystalline cellulose, enhances the hydrolytic activity of cellulase hydrolases (Vaaje-Kolstad et al., 2010). GH18 catalyzes the biodegradation of the *β*-1,4 glycosidic bond in aminoglycans through a substrate-assisted retention mechanism, and is involved in various physiological processes, including tissue degradation and remodeling, nutrient uptake, invasion, and pathogenesis, as well as immune response regulation (Sørbotten et al., 2005). The enrichment of genes encoding all of the above-mentioned CAZymes in *Sanghuangporus* highlights their importance in lignocellulose degradation, and it can be assumed that these genes are core genes required for efficient nutrient utilization. Meanwhile, our data provide a theoretical basis for screening and utilization of this genetic resource, to breed new varieties of *Sanghuangporus* with excellent qualities. Certain CAZyme families, including GTs, are encoded by more genes in *S. weigelae* than in *S. baumii* and *S. sanghuang* (**Figure 4**), indicating that *S. weigelae* has unique nutritional strategy that warrants further study.

CYPs are an important class of monooxygenases with vital roles in various biological activities (Črešnar and Petrič, 2011; Zeng et al., 2016); however, fungal CYPs have been little studied relative to those of mammals and plants (Črešnar and Petrič, 2011; Qhanya et al., 2015). In the current study, 23 of 130 CYPs identified in *S. weigelae*, compared with 18 of 112 CYPs in *S. baumii*, 25 of 121 CYPs in *S. sanghuang*, and 24 of 136 CYPs in *S. vaninii* could not be categorized into any known class (**Table 3**). It was speculated that the 23 genes might participate in several types of secondary metabolic processes, including biodegradation of xenobiotics, carbohydrate metabolism, and biosynthesis of antibiotics. Furthermore, there are specific types of CYPs in basidiomycetous biotrophic plant pathogens that allow the mushrooms to adapt to a wide range of ecological niches. Therefore, these 23 *S. weigelae* specific CYPs may be involved in important adaptive mechanisms specific to the host plant. In addition, similar uncharacterized CYPs were detected in *S. baumii*, *S. vaninii*, and *S. sanghuang*, and hypothesized to be associated with host adaptation. Functional experiments are required to clarify the roles of these genes in *Sanghuangporus* evolution and their relationship with host adaptation. In future, comparative transcriptomics between *Sanghuangporus* species at the primordium and fruiting body formation stages will reveal the key genes involved in these processes and thus facilitate cultivation of *S. weigela*e.

## 5 Conclusions

Here, we generated the first whole genome sequence of medicinal mushroom *S. weigelae*, represented by a monokaryotic strain. Integrity, completeness, and collinearity analyses revealed the high quality of our genome assembly. Comparative genomic and phylogenetic analyses indicated clearly that *S. weigelae* should be classified in the *Sanghuangporus* genus. In addition, identification of CAZyme-encoding genes revealed that *S. weigelae* has robust lignocellulose degradation capacity. Overall, the *S. weigelae* genome provides insights useful for basic research into the nutrition and medicinal utility of this mushroom. Comprehensive understanding of the *S. weigelae* genome has potential to provide a basis for its future application in pharmacological and industrial fields. Future generation of transcriptomic and metabolic data will further facilitate the appropriate application of *S. weigelae*.

## Data availability statement

The datasets presented in this study can be found in online repositories. The names of the repository/repositories and accession number(s) can be found below: BioProject PRJNA1030531.

## Author contributions

CJ: Data curation, Formal Analysis, Investigation, Methodology, Software, Validation, Visualization, Writing – original draft, Writing – review & editing. JM: Formal Analysis, Investigation, Software, Validation, Writing – original draft, Writing – review & editing. HW: Investigation, Validation, Writing – original draft. LT: Investigation, Validation, Writing – original draft. YY: Investigation, Validation, Writing – original draft. XL: Investigation, Validation, Writing – original draft. JS: Conceptualization, Data curation, Formal Analysis, Funding acquisition, Investigation, Methodology, Project administration, Resources, Supervision, Validation, Writing – original draft, Writing – review & editing.
