# Supplementary material for: First genome assembly and annotation of Sanghuangporus weigelae uncovers its medicinal functions, metabolic pathways, and evolution
Source: Front Cell Infect Microbiol. 2024 Jan 9;13:1325418. doi: 10.3389/fcimb.2023.1325418 (PMC10803629; doi:10.3389/fcimb.2023.1325418)
Supplement: Supplementary file 1 [file DataSheet_1.doc]

**Table S1 Data on genome sequences.**

| **Phylum** | **Species** | **Voucher** | **BioProject ID** | **Assembly level** | **Genome size (Mb)** | **Numbers** | **Scaffold N50** | **GC (%)** |
| --- | --- | --- | --- | --- | --- | --- | --- | --- |
| 1 | *Neurospora crassa* | OR74A | PRJNA13841 | chromosome | 41 | 7 | 6 Mb | 48 |
| 2 | *Tuber melanosporum* | Mel28 | PRJEA38847 | Scaffold | 124.9 | 398 | 639 kb | 44.5 |
| 3 | *Agaricus bisporus* | H97 | PRJNA61005 | Scaffold | 30.2 | 29 | 2.3 Mb | 46 |
| 4 | *Auricularia subglabra* | TFB-10046 SS5 | PRJNA60553 | Scaffold | 74.9 | 1531 | 491.4 kb | 58.5 |
| 5 | *Fomitiporia mediterranea* | MF3 / 22 | PRJNA56107 | Scaffold | 63.4 | 1412 | 4.3 Mb | 40.5 |
| 6 | *Ganoderma sinense* | ZZ0214-1 | PRJNA42807 | Scaffold | 49 | 69 | 2.3 Mb | 56 |
| 7 | *Hericium alpestre* | DSM 108284 | PRJNA521006 | Scaffold | 37.5 | 3534 | 16.9 kb | 52.5 |
| 8 | *Heterobasidion irregulare* | TC 32-1 | PRJNA46703 | Scaffold | 33.6 | 15 | 2.6Mb | 52 |
| 9 | *Phellinidium pouzarii* | DSM 108285 | PRJNA521454 | contig | 28.6 | 1776 | 41.4 kb | 47 |
| 10 | *Pleurotus ostreatus* | PC15 | PRJNA81933 | chromosome | 35.3 | 11 | 3.3 Mb | 51 |
| 11 | *Polyporus arcularius* | HHB13444 | PRJNA196048 | Scaffold | 43.5 | 2540 | 48.8 kb | 57 |
| 12 | *Pyrrhoderma noxium* | FFP RI411160 | PRJNA377805 | Chromosome | 31.4 | 12 | 3.4 Mb | 41.5 |
| 13 | *Rickenella mellea* | SZMC22713 | PRJNA334780 | Scaffold | 45.1 | 848 | 362 kb | 48.5 |
| 14 | *Sanghuangporus baumii* | 821 | PRJNA304358 | Scaffold | 31.64 | 217 | 267.1 kb | 47.25 |
| 15 | *Sanghuangporus sanghuang* | MS2 | PRJNA731269 | Scaffold | 33.34 | 26 | 2.06 Mb | 48.04 |
| 16 | *Sanghuangporus vaninii* | Kangneng | PRJNA564179 | Scaffold | 34.52 | 11310 | 9.3 kb | 47.95 |
| 17 | *Sanghuangporus weigelae* | Si20 | PRJNA1030531 | Chromosome | 33.9 | 13 | 2.77 Mb | 47.93 |
| 18 | *Schizophyllum commune* | H4-8 | PRJNA32757 | Scaffold | 38.7 | 25 | 2.6 Mb | 57.5 |
| 19 | *Schizopora paradoxa* | KUC8140 | PRJNA239088 | Scaffold | 44.4 | 1291 | 121.5 kb | 49 |
| 20 | *Trametes versicolor* | FP-101664 SS1 | PRJNA56097 | Scaffold | 44.8 | 283 | 2.9 Mb | 57.5 |
| 21 | *Wolfifiporia cocos* | MD-104 | PRJNA52943 | contig | 64.4 | 76 | 3.8 Mb | 51.5 |
| 22 | *Leucocoprinus birnbaumii* | VT141 | PRJNA862647 | Scaffold | 45.7 | 2238 | 32 kb | 47.5 |
| 23 | *Gymnopus androsaceu* | JB14 | PRJNA234428 | Scaffold | 89.1 | 2516 | 171.6 kb | 45 |

**Table S2 Genes involved in terpenoid backbone biosynthesis pathway in *Sanghuangporus weigelae*.**

| **Gene name and definition** | **Enzyme code** | **KO term** | **Gene ID** |
| --- | --- | --- | --- |
| FDPS; farnesyl diphosphate synthase | 2.5.1.1, 2.5.1.10 | K00787 | C06.600 |
| mvaD; diphosphomevalonate decarboxylase | 4.1.1.33 | K01597 | C05.149 |
| FNTA; protein farnesyltransferase / geranylgeranyltransferase type‐1 subunit *α* | 2.5.1.58 | K05955 | C08.369 |
| STE24; STE24 endopeptidase | 3.4.24.84 | K06013 | C11.293 |
| HMGCR; hydroxymethylglutaryl‐CoA reductase (NADPH) | 1.1.1.34 | K00021 | C13.33 |
| atoB; acetyl‐CoA C‐acetyltransferase | 2.3.1.9 | K00626 | C04.483 |
| GGPS1; geranylgeranyl diphosphate synthase type III | 2.5.1.1,  2.5.1.10,2.5.1.29 | K00804 | C09.675 |
| mvaK2; phosphomevalonate kinase | 2.7.4.2 | K00938 | C05.66 |
| E.2.3.3.10; hydroxymethylglutaryl‐CoA synthase | 2.3.3.10 | K01641 | C02.532 |
| idi; isopentenyl‐diphosphate *γ*‐isomerase | 5.3.3.2 | K01823 | C01.906 |
| hexPS; hexaprenyl‐diphosphate synthase | 2.5.1.82, 2.5.1.83 | K05355 | C01.249 |
| FCLY; prenylcysteine oxidase / farnesylcysteine lyase | 1.8.3.5, 1.8.3.6 | K05906 | C01.1008 |
| FNTB; protein farnesyltransferase subunit *β* | 2.5.1.58 | K05954 | C11.353 |
| SRT1; ditrans polycis‐polyprenyl diphosphate synthase | 2.5.1.87 | K11778 | C03.301 |
| RCE1 FACE2; prenyl protein peptidase | 3.4.22.-(FACE2) | k08658 | C12.184 |
| ICMT STE14; protein-*S*-isoprenylcysteine *O*-methyltransferase | 2.1.1.100 | k00587 | C04.371  C09.365  C09.368 |

**Table S3 Genes involved in polysaccharide (starch and sucrose) biosynthesis in *Sanghuangporus weigelae*.**

| **Gene name and definition** | **Enzyme code** | **KO term** | **Gene ID** | **Identity** | **E value** |
| --- | --- | --- | --- | --- | --- |
| HK; hexokinase | 2.7.1.1 | k00844 | C04.422 | 42.331 | 1.30E-115 |
| HK; hexokinase | 2.7.1.1 | k00844 | C12.2 | 46.078 | 4.79E-144 |
| malZ; *α*-glucosidase | 3.2.1.20 | K01187 | C01.1384 | 41.244 | 0 |
| EGLC; glucan endo-1,3-*β*-_D_-glucosidase | 3.2.1.39 | K01199 | C10.151 | − | 8.50E-124 |
| EGLC; glucan endo-1,3-*β*-_D_-glucosidase | 3.2.1.39 | K01199 | C10.156 | − | 6.80E-83 |
| Glucan 1,3-*β*-glucosidase | 3.2.1.58 | k01210 | C01.236 | 47.25 | 1.80E-112 |
| Glucan 1,3-*β*-glucosidase | 3.2.1.58 | k01210 | C01.241 | 46.97 | 3.03E-110 |
| Glucan 1,3-*β*-glucosidase | 3.2.1.58 | k01210 | C03.560 | 36.364 | 7.50E-84 |
| Glucan 1,3-*β*-glucosidase | 3.2.1.58 | k01210 | C05.760 | 33.105 | 6.68E-78 |
| Glucan 1,3-*β*-glucosidase | 3.2.1.58 | k01210 | C06.584 | 33.014 | 1.70E-21 |
| *β*-glucosidase | 3.2.1.21 | k01188 | C02.982 | 65.598 | 0 |
| *β*-glucosidase | 3.2.1.21 | k01188 | C12.140 | 70.611 | 0 |
| bglX; *β*-glucosidase | 3.2.1.21 | K05349 | C01.801 | 52.697 | 0 |
| bglX; *β*-glucosidase | 3.2.1.21 | K05349 | C03.431 | 43.649 | 0 |
| bglX; *β*-glucosidase | 3.2.1.21 | K05349 | C03.884 | 43.438 | 0 |
| bglX; *β*-glucosidase | 3.2.1.21 | K05349 | C04.1015 | 32.357 | 1.58E-101 |
| bglX; *β*-glucosidase | 3.2.1.21 | K05349 | C10.113 | 61.079 | 0 |
| bglX; *β*-glucosidase | 3.2.1.21 | K05349 | C10.370 | 50.949 | 0 |
| bglX; *β*-glucosidase | 3.2.1.21 | K05349 | C10.372 | 51.626 | 0 |
| GPI, pgi; glucose-6-phosphate isomerase | 5.3.1.9 | k01810 | C13.331 | 79.151 | 0 |
| Endoglucanase | 3.2.1.4 | k001179 | C01.476 | 72.067 | 0 |
| Endoglucanase | 3.2.1.4 | k001179 | C04.769 | 36.831 | 4.48E-75 |
| Endoglucanase | 3.2.1.4 | k001179 | C07.30 | 58.398 | 1.19E-156 |
| Endoglucanase | 3.2.1.4 | k001179 | C07.54 | 54.072 | 1.35E-114 |
| CBH1; cellulose 1,4-*β*-cellobiosidase | 3.2.1.91 | k01225 | C11.87 | 64.035 | 0 |
| CBH1; cellulose 1,4-*β*-cellobiosidase | 3.2.1.91 | k01225 | C02.695 | 68.502 | 0 |
| CBH1; cellulose 1,4-*β*-cellobiosidase | 3.2.1.91 | k01225 | C02.694 | − | 2.90E-276 |
| CBH1; cellulose 1,4-*β*-cellobiosidase | 3.2.1.91 | k01225 | C02.685 | 67.841 | 0 |
| CBH2, cbhA; cellulose1,4-*β*-cellobiosidase | 3.2.1.91 | k19668 | C01.218 | 60.623 | 3.70E-151 |
| CBH2, cbhA; cellulose1,4-*β*-cellobiosidase | 3.2.1.91 | k19668 | C03.338 | 65.393 | 0 |
| 1,3-*β*-glucan synthase | 2.4.1.34 | k00706 | C03.688 | 70.641 | 0 |
| 1,3-*β*-glucan synthase | 2.4.1.34 | k00706 | C12.48 | 73.659 | 0 |
| pgm; phosphoglucomutase | 5.4.2.2 | k01835 | C02.598 | 58.467 | 0 |
| pgm; phosphoglucomutase | 5.4.2.2 | k01835 | C03.442 | 45.47 | 1.06E-179 |
| UGP2, galU, galF; UTP-glucose-1-phosphate-uridylyltransferase | 2.7.7.9 | k00963 | C05.222 | 59.756 | 0 |
| otsA; trehalose 6-phosphate synthase | 2.4.1.15 | k00697 | C07.182 | 64.286 | 0 |
| TPS; trehalose 6-phosphatesynthase / phosphatase | 2.4.1.15, 3.1.3.12 | K16055 | C08.249 | 39.394 | 0 |
| TSL1, TPS3; trehalose 6-phosphate synthase complex regulatory subunit | 2.4.1.15 | k22337 | C10.358 | 34.839 | 7.29E-144 |
| GYG1, GYG2; glycogenin | 2.4.1.11,2.4.1.186 | k00750 | C01.384 | 40.351 | 1.19E-46 |
| GYS; glycogen synthase | 2.4.1.11 | K00693 | C11.65 | 70.499 | 0 |
| PYG, glgP; glycogen phosphorylase | 2.4.1.1 | K00688 | C10.201 | 52.673 | 0 |
| AGL; glycogen debranching enzyme | 2.4.1.1 | K01196 | C02.192 | 42.711 | 0 |
| TSL1, TPS3; trehalose 6-phosphate synthase complex regulatory subunit | 3.1.3.12 | K22337 | C10.358 | − | 0 |
| TPS; trehalose 6-phosphate synthase / phosphatase | 3.1.3.12 | K16055 | C08.249 | − | 0 |
| GBE1, glgB; 1,4-*α*-glucan branching enzyme | 2.4.1.18 | K00700 | C04.450 | 73.383 | 0 |
| AMY, amyA, malS; *α*-amylase | 3.2.1.1 | K01176 | C03.388 | 44.311 | 1.60E-130 |
| AMY, amyA, malS; *α*-amylase | 3.2.1.1 | K01176 | C07.332 | 36.893 | 2.79E-107 |
| AMY, amyA, malS; *α*-amylase | 3.2.1.1 | K01176 | C10.410 | 43.34 | 1.12E-129 |
| TREH, treA, treF; *α*,*α*-trehalase | 3.2.1.28 | K01194 | C07.204 | 54.953 | 0 |
| TREH, treA, treF; *α*,*α*-trehalase | 3.2.1.28 | K01194 | C09.129 | 35.614 | 1.14E-93 |

**Table S4 Putative genes involved in flavonoid biosynthesis in *Sanghuangporus weigelae* with *Arabidopsis* as reference.**

| **Gene name and definition** | **Enzyme code** | **KO term** | **Gene ID** | **Identity** | **E-value** |
| --- | --- | --- | --- | --- | --- |
| P-type H^+^-ATPase | 3.6.3.6 | K01535 | C08.242 | 53.077 | 0 |
| Anthocyanin 3-*O*-6″-*O*-coumaroylglucoside: glucosyltransferase | 3.2.1.21 | K01188 | C02.982 | 42.484 | 1.36E-118 |
| Anthocyanin 3-*O*-6″-*O*-coumaroylglucoside: glucosyltransferase | 3.2.1.21 | K01188 | C12.140 | 40.417 | 4.93E-112 |
| Acetyl-CoA carboxylase | 2.1.3.15 | K11262 | C02.64 | 40.304 | 0 |
| Glutathione S-transferase | 2.5.1.18 | K00799 | C01.994 | 40.094 | 4.34E-43 |
| Flavonoid 3-*O*-glucosyltransferase | 3.2.1.21 | K05349 | C04.1015 | 35.878 | 1.98E-17 |


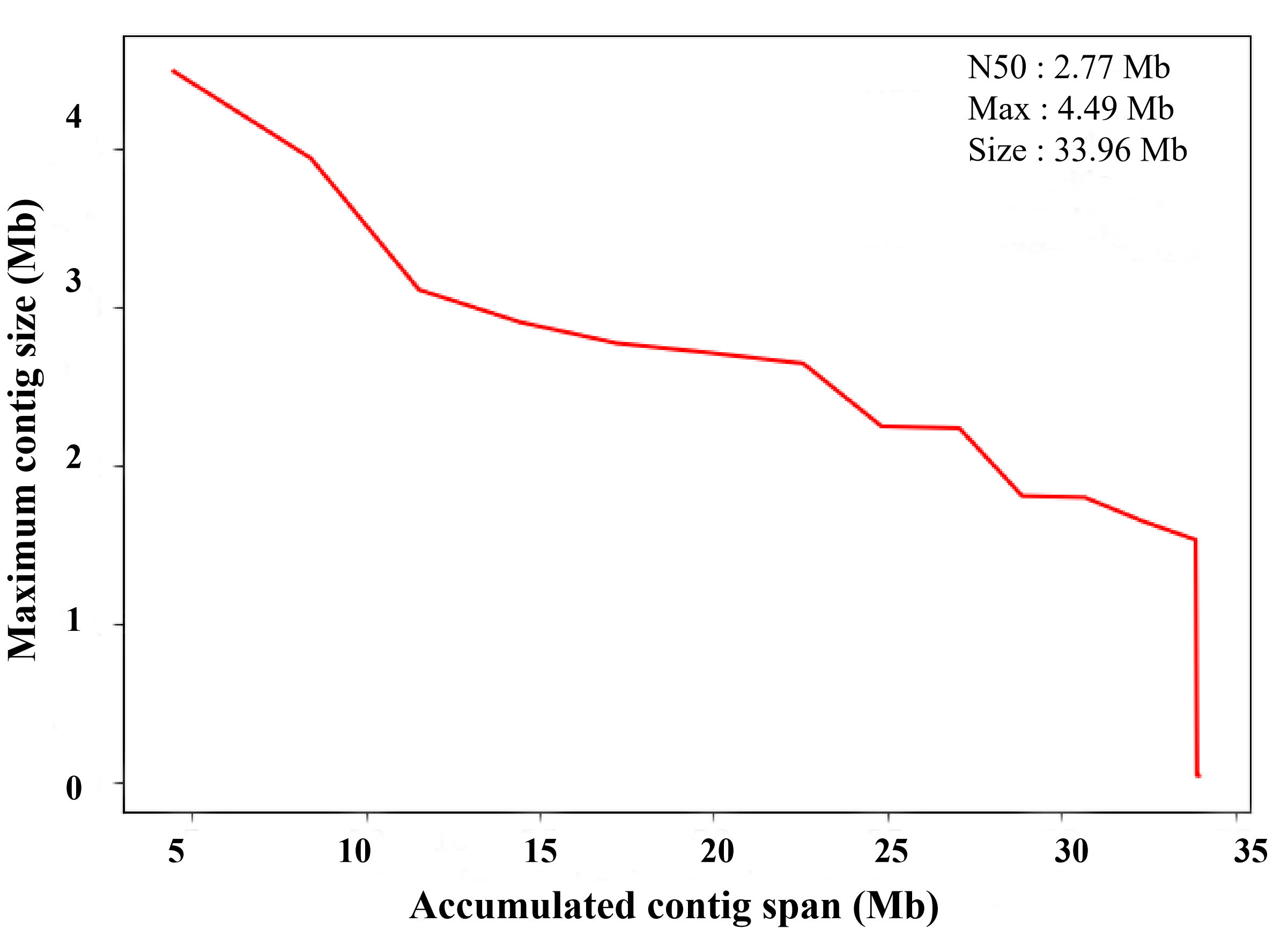


**Figure S1 Contig length cumulative graph of *Sanghuangporus weigelae* genome.**


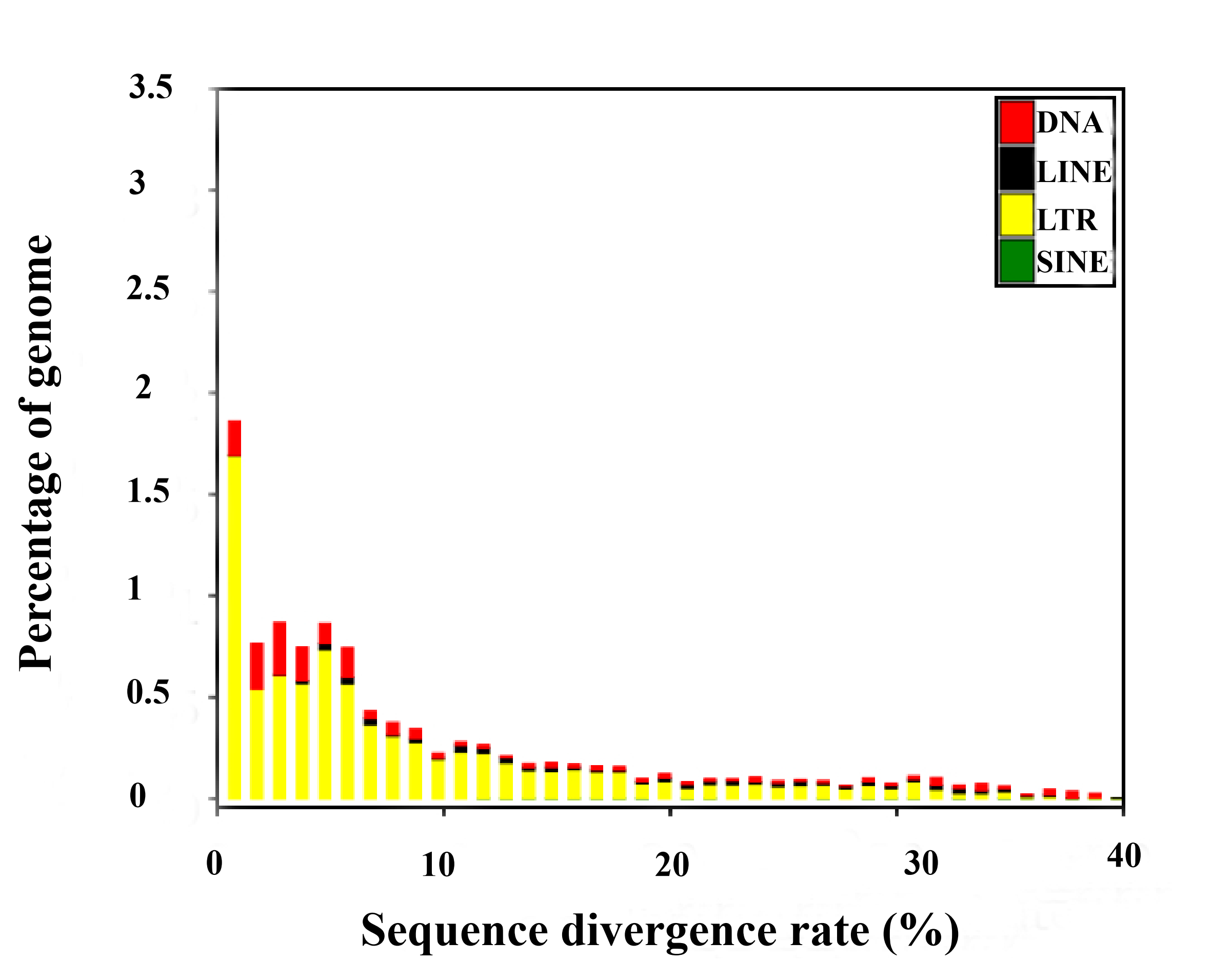


**Figure S2 Transposon differentiation rate of *Sanghuangporus weigelae* genome.**


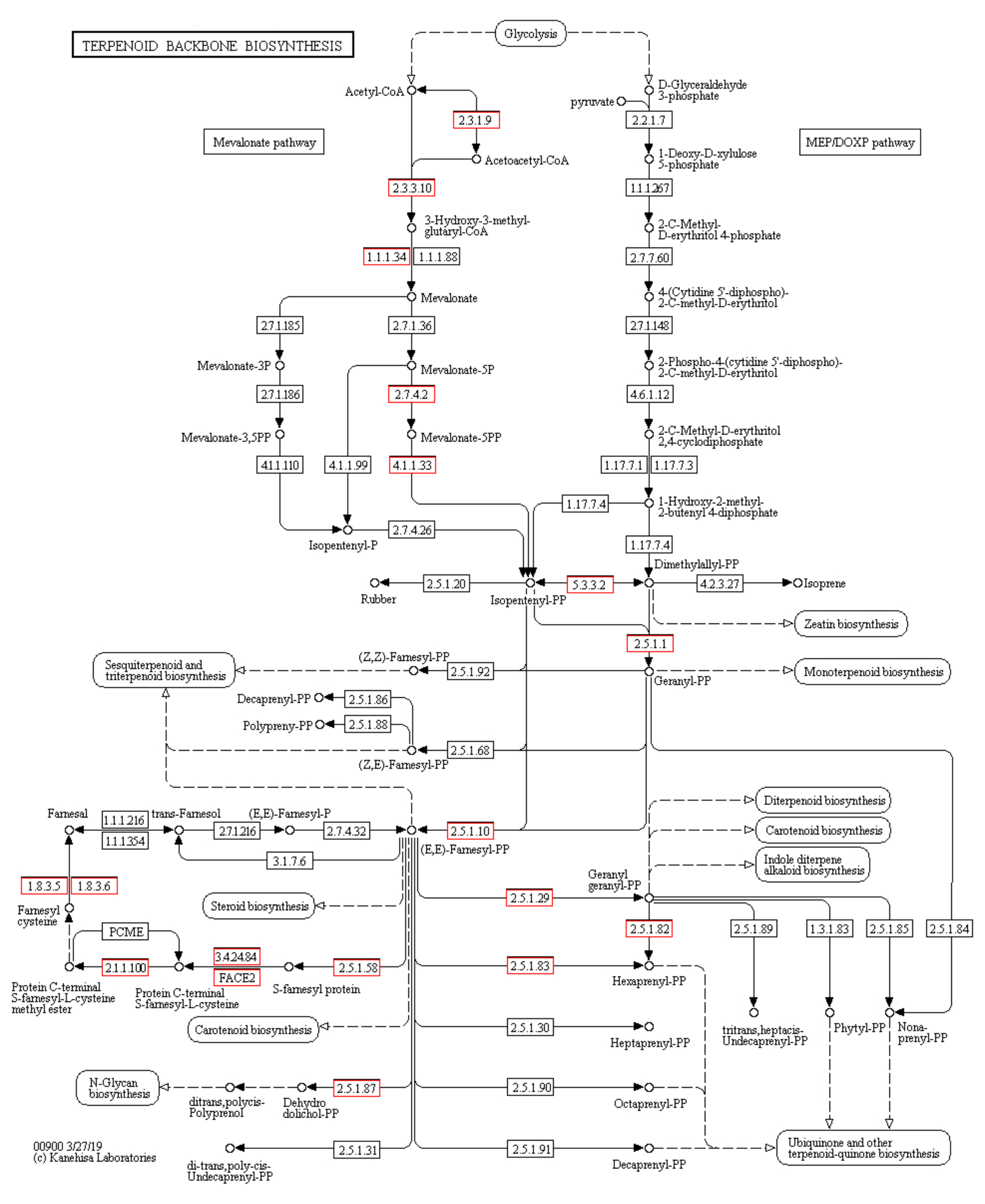


**Figure S3 Terpenoid backbone biosynthesis pathways in *Sanghuangporus weigelae*.**


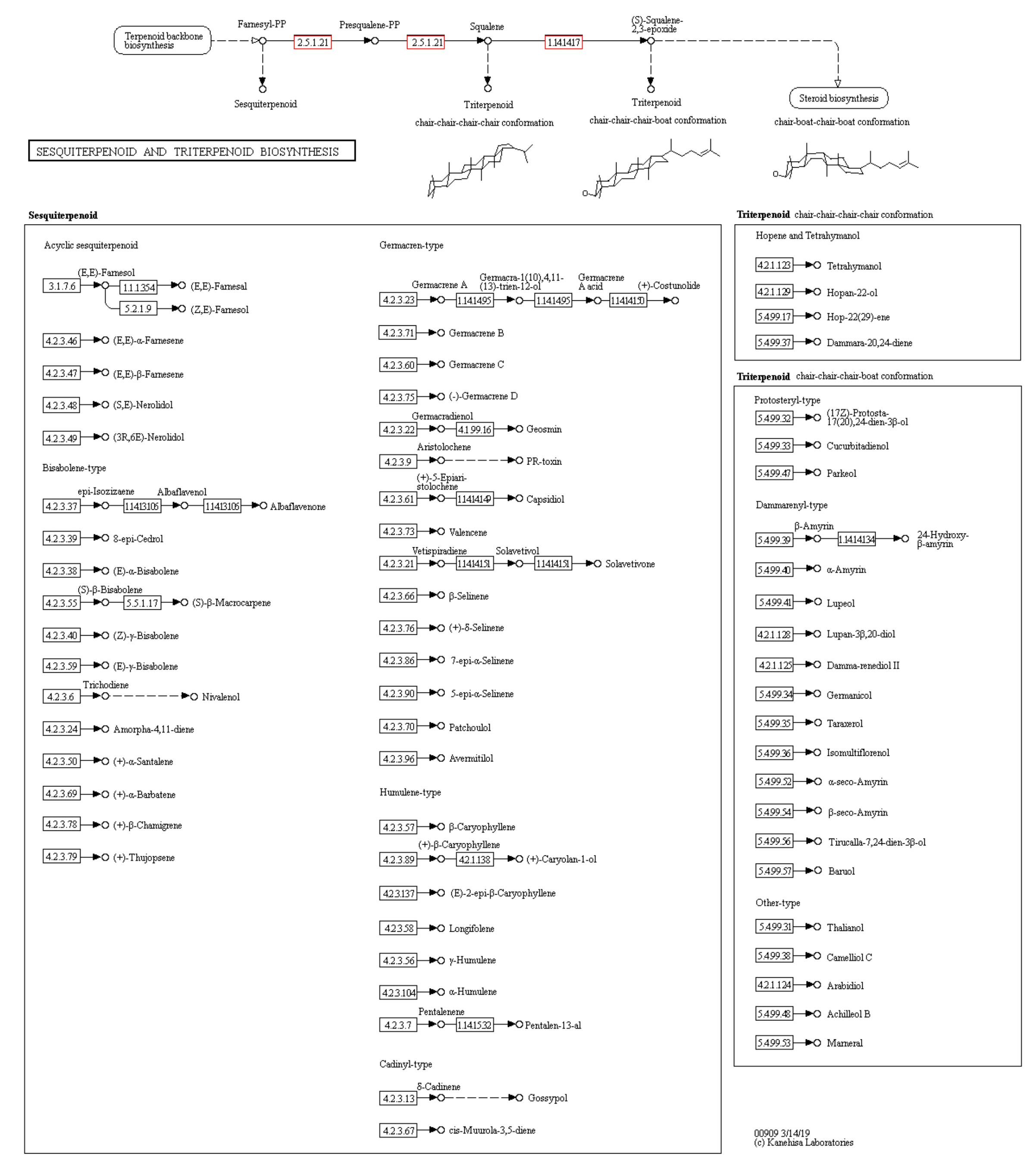


**Figure S4 Sesquiterpenoid and triterpenoid biosynthesis pathways in *Sanghuangporus weigelae*.**


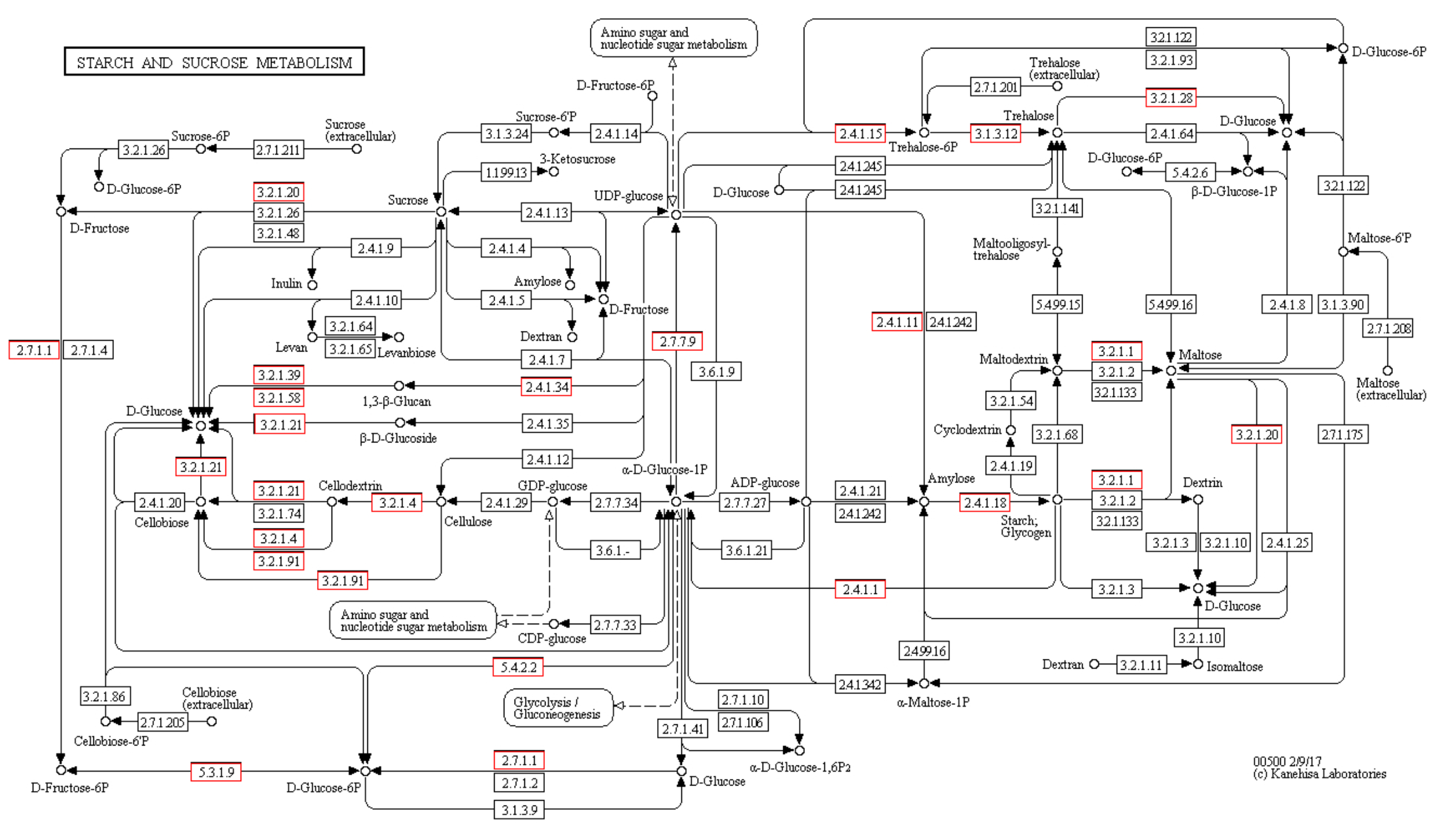


**Figure S5 Polysaccharide (starch and sucrose) biosynthesis pathways in *Sanghuangporus weigelae*.**


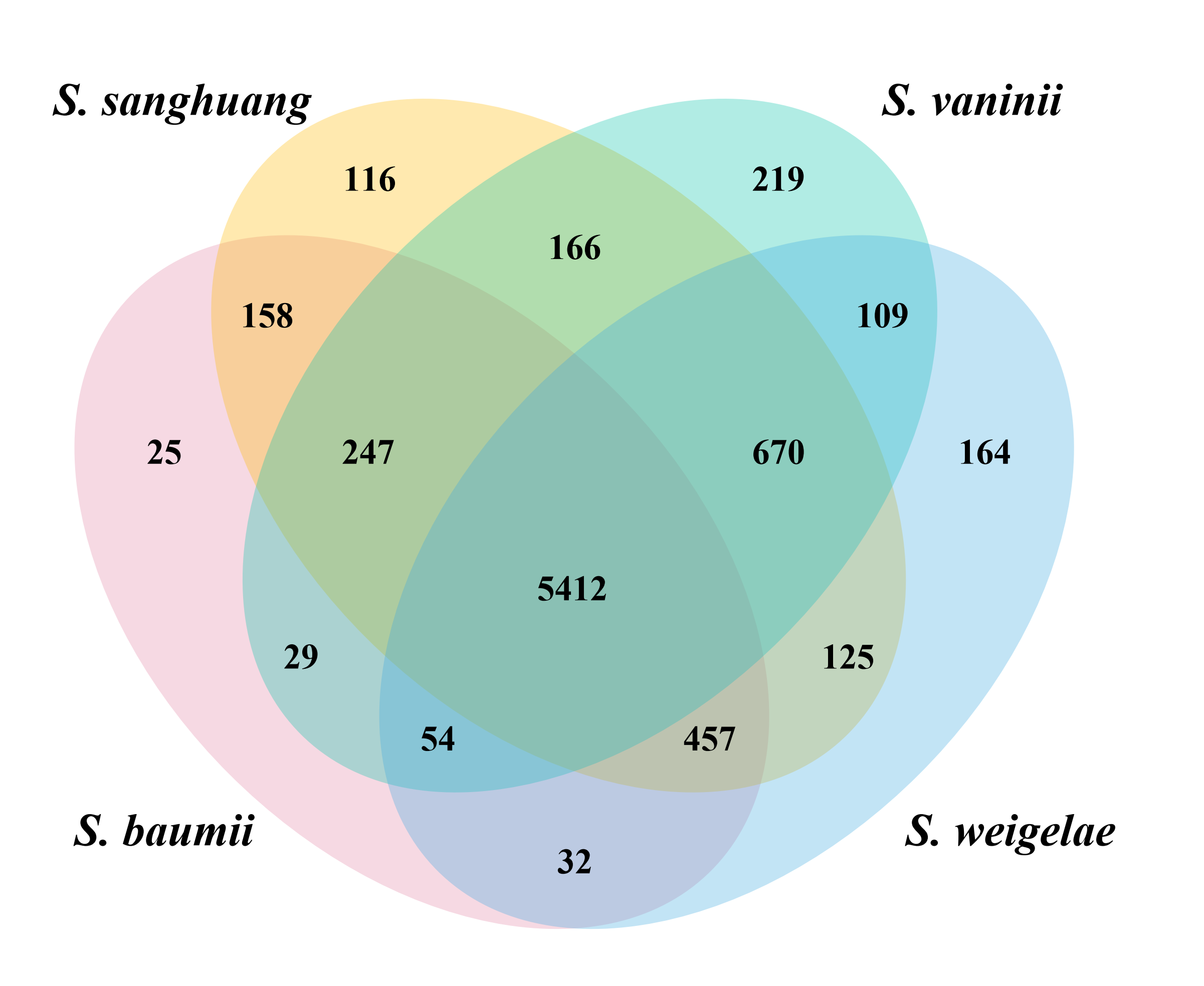


**Figure S6 Venn diagram of the orthogroups of four *Sanghuangporus* species.**
